# Defining the Product Chemical Space of Monoterpenoid Synthases

**DOI:** 10.1371/journal.pcbi.1005053

**Published:** 2016-08-12

**Authors:** Boxue Tian, C. Dale Poulter, Matthew P. Jacobson

**Affiliations:** 1 Department of Pharmaceutical Chemistry, School of Pharmacy, University of California, San Francisco, San Francisco, California, United States of America; 2 California Institute for Quantitative Biomedical Research, University of California, San Francisco, San Francisco, California, United States of America; 3 Department of Chemistry, University of Utah, Salt Lake City, Utah, United States of America; Wake Forest University, UNITED STATES

## Abstract

Terpenoid synthases create diverse carbon skeletons by catalyzing complex carbocation rearrangements, making them particularly challenging for enzyme function prediction. To begin to address this challenge, we have developed a computational approach for the systematic enumeration of terpenoid carbocations. Application of this approach allows us to systematically define a nearly complete chemical space for the potential carbon skeletons of products from monoterpenoid synthases. Specifically, 18758 carbocations were generated, which we cluster into 74 cyclic skeletons. Five of the 74 skeletons are found in known natural products; some of the others are plausible for new functions, either in nature or engineered. This work systematizes the description of function for this class of enzymes, and provides a basis for predicting functions of uncharacterized enzymes. To our knowledge, this is the first computational study to explore the complete product chemical space of this important class of enzymes.

## Author Summary

Terpenoids, as one of the largest classes of natural products, provide complex carbocycle structures for many drugs (e.g. taxol) and prodrugs. The diverse carbocycle structures arise from complex carbocation rearrangements catalyzed by terpenoid synthases. Many putative terpene synthase enzymes identified in genome sequencing efforts remain functionally uncharacterized, and some of these will undoubtedly have novel products, potentially including previously undiscovered carbocycles. In this work, we present a computational approach that systematically enumerates all plausible carbocations of monoterpenoid synthases in order to define and organize the potentially large product chemical space of this important class of enzymes.

## Introduction

**Fig 1:**
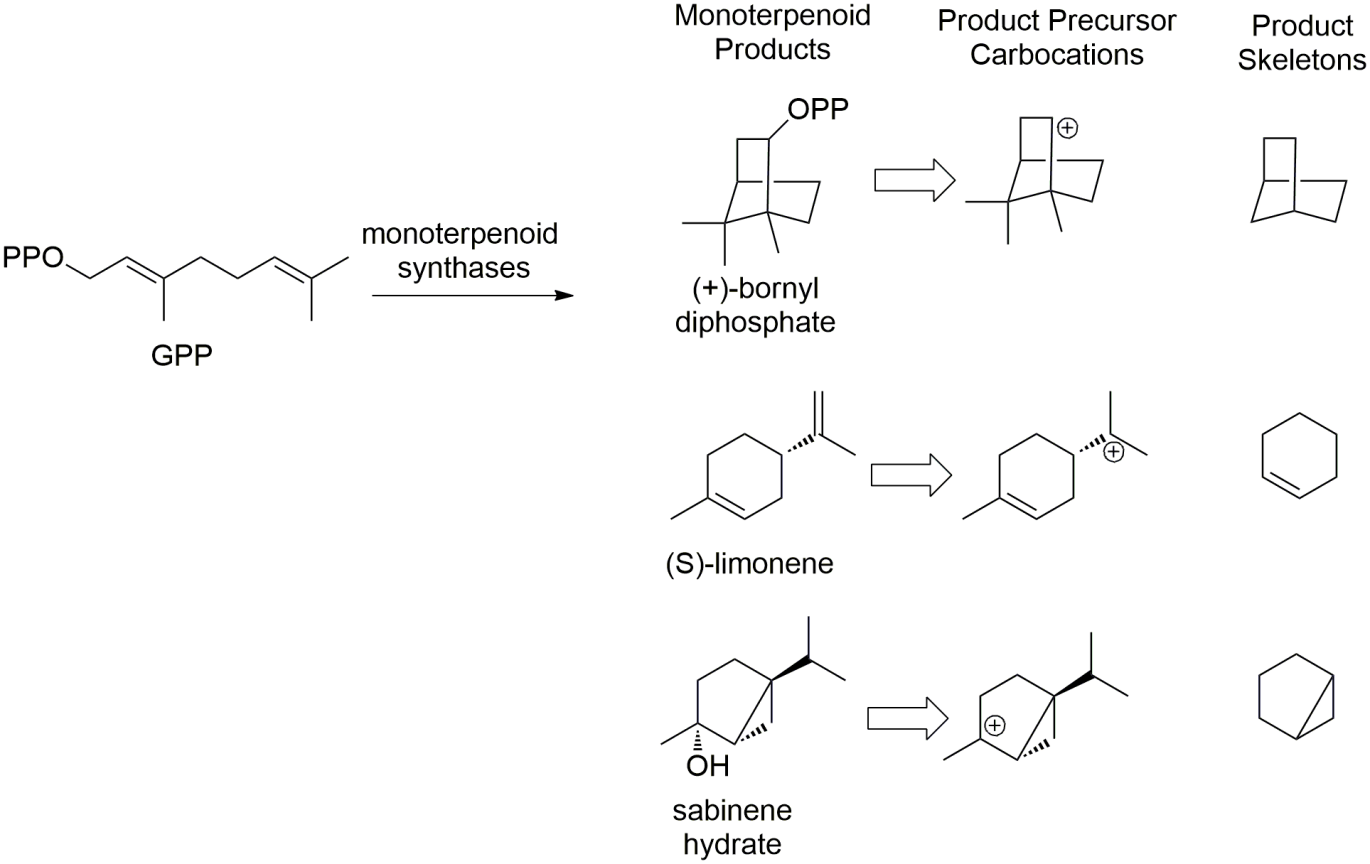
Example monoterpene compounds, their carbocation precursors, and skeletons. Product precursor carbocations are quenched by phosphorylation, deprotonation, or hydration to yield products.

Terpenoids, which have diverse carbon skeletons, are an important class of natural products [1–3]. To date, more than 63,000 different terpenoids have been reported [4]. In nature, most cyclic terpenoids are created by terpenoid synthases (sometimes called terpenoid cyclases [5]), which catalyze the cyclizations of linear terpenes such as geranyl diphosphate through carbocation rearrangements [6]. The cyclized carbocationic intermediates are ultimately quenched by phosphorylation, deprotonation, or hydration to yield products (Fig 1). The intrinsic reactivity of carbocations plays an important role in the outcome of cyclization [7–9]. Terpenoids are classified as monoterpenes (C_10_), sesquiterpenes (C_15_), diterpenes (C_20_), sesterterpenes (C_25_), triterpenes (C_30_) and sesquarterpenes (C_35_) according to the number of C_5_ isoprenoid units incorporated into their carbon skeletons.

Rapid advances in DNA sequencing provide an opportunity to discover enzymes involved in creating both previously characterized and novel terpenoid natural products. The gap between sequenced genes and reliable functional annotations is enormous and increasing. For example, the Structure-Function Linkage Database (version 2014) [10] assigns 2778 enzyme sequences to the terpene synthase subgroup of the isoprenoid synthase 2 superfamily (Mg-dependent), of which 2540 (91%) are annotated as having ‘unknown’ function. Thus, the functions of the large majority of these enzymes remain uncharacterized.

Inferring enzyme function from protein sequence is challenging in general [11], and is likely to be particularly difficult for enzymes involved in terpenoid biosynthesis, because 1) the potential product chemical space is huge, and 2) single point mutations can alter product specificity [12]. In previous work, we have predicted enzyme substrates and products from protein sequence by using a combination of bioinformatics and structural modeling [13,14]. In order to apply similar methods to terpene synthases, a first major challenge is simply to enumerate the possible enzyme activities that could exist among the uncharacterized enzymes. Defining the possible substrates is trivial (C_5_, C_10_, C_15_, etc.), although there have been investigations into the catalytic mechanisms of a few terpene synthases [6], no previous attempts have been made to systematically define the possible products, due to the complexity of the problem.

In this work, we systematically enumerate thousands of potential monoterpenoid carbocationic intermediates, by using computer simulations. To present the complex results in a simple manner, we organize the carbocationic intermediates according to their cyclic ring structures and the locations of double bonds within the carbocycles. We identify 74 such cyclic product skeletons, among which (at least) 5 are represented among characterized monoterpenoid natural products. Among the remaining skeletons, several appear to be plausible albeit hypothetical monoterpene skeletons, in the sense that they can be connected to the linear substrate by a relatively small number of carbocation rearrangements known to occur in terpene synthases. Thus, although natural products with these skeletons do not appear to have been reported, they may be found among the products of the many currently uncharacterized terpene synthases, or be accessible via enzyme engineering.

## Results

### Automatic enumeration of carbocations

**Fig 2:**
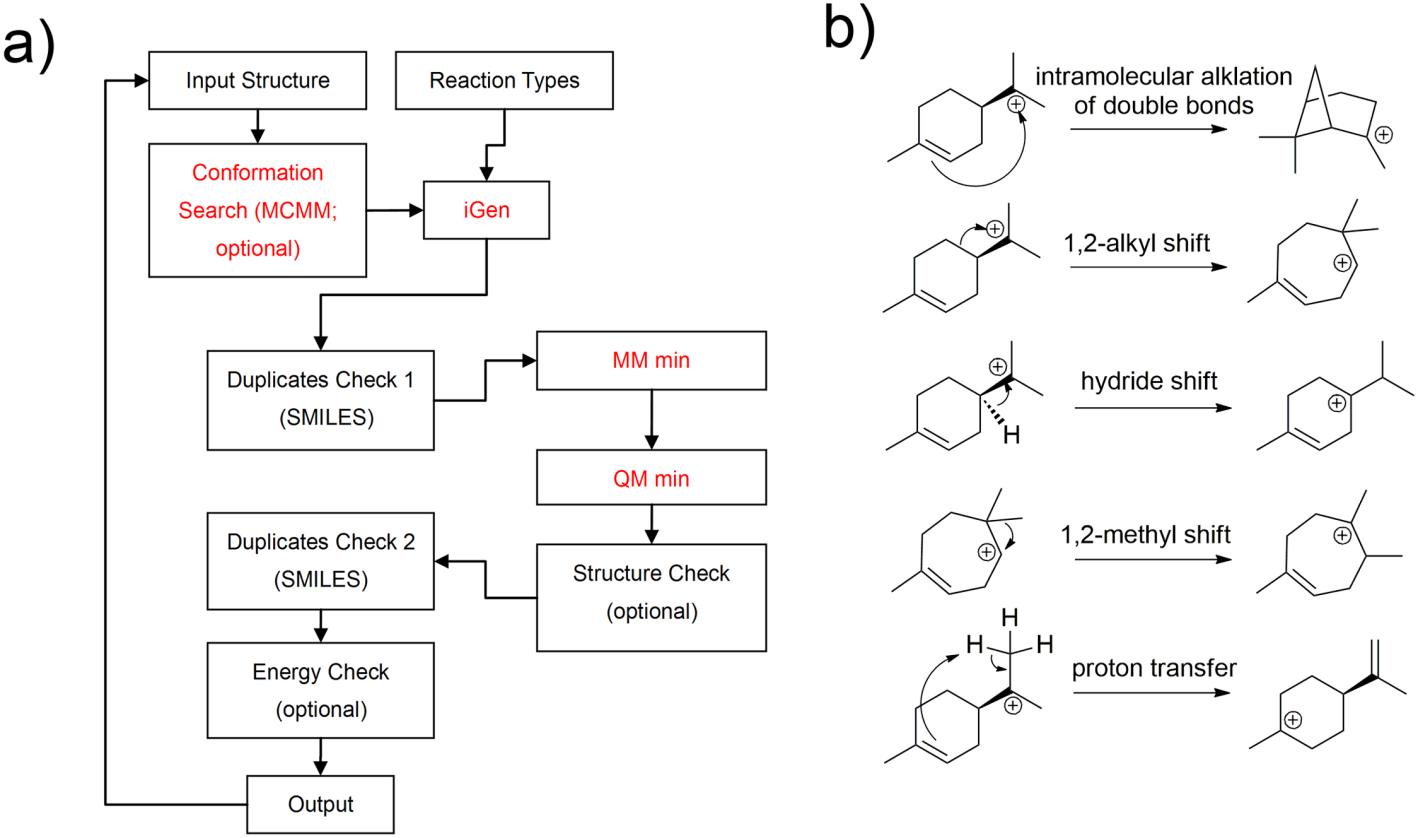
The iGen algorithm. (a) Schematic overview. Red modules can be run in parallel on multiple computer cores. (b) Reaction types applied to the carbocations, obtained from mechanistic studies of terpenoid synthases.

**Fig 3:**
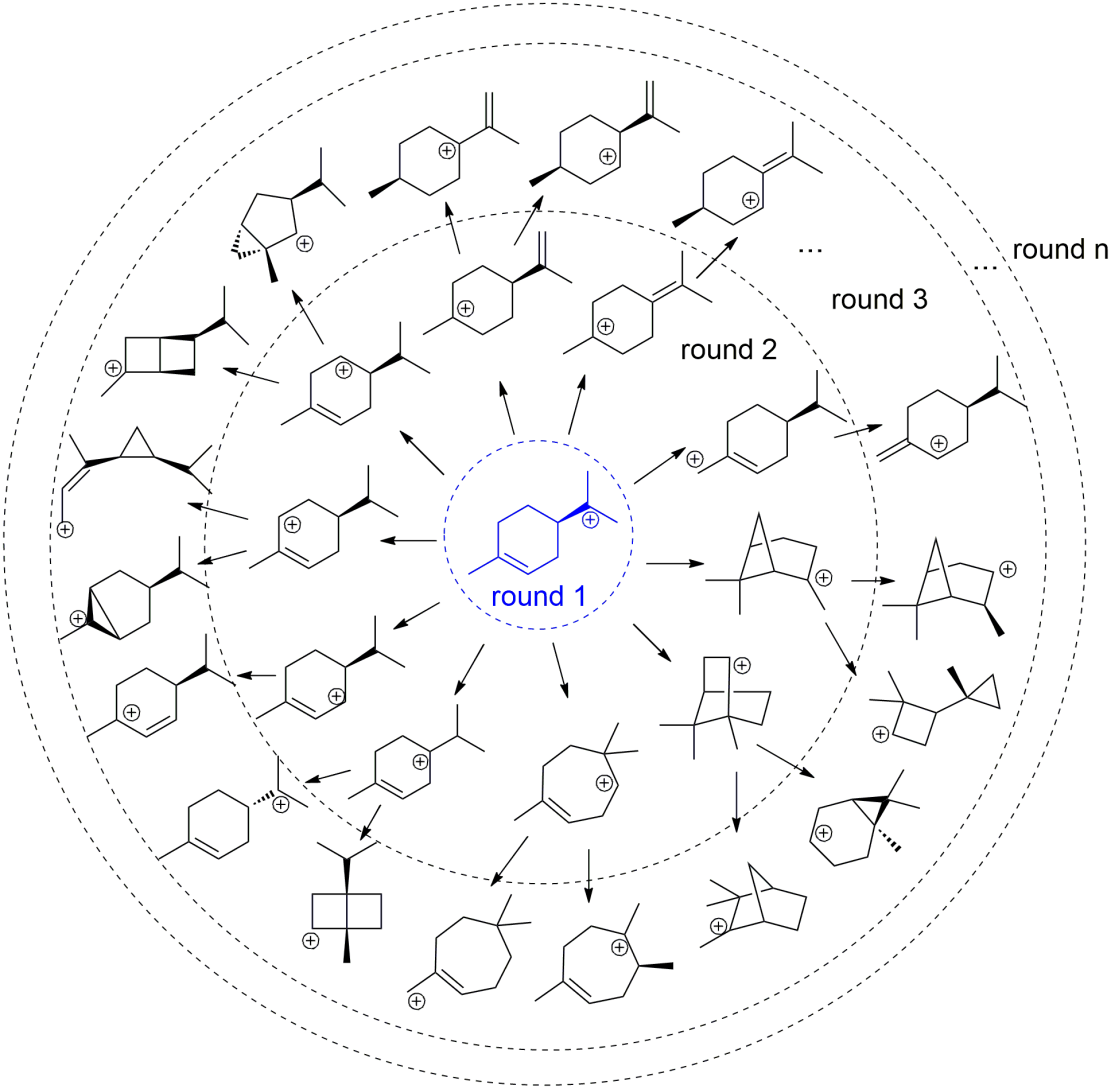
Example output of iGen, starting from one cyclized carbocation.

Our simulations perform virtual carbocation rearrangements in the gas phase (Figs 2 and 3 and S1 Movie), allowing the enumeration of all carbocations that follow from cyclization of the linear allylic monoterpene carbocation. Five reaction types are considered (Fig 2b): 1) intramolecular alkylation of double bonds; 2) alkyl shifts (excluding 1,2-methyl shifts); 3) hydride shifts; 4) 1,2-methyl shifts; 5) proton transfers. All five types of reactions were carried out for each carbocationic intermediate (details see Methods). The energies of product carbocations were evaluated by semi-empirical quantum mechanics to ensure their thermo-stability at room temperature (0 kcal/mol relative energy filter, see Methods). The ‘Simplified Molecular Input Line Entry System’ (SMILES), which describes the chemical structures using ASCII strings (Fig 2a; details see Methods), is used to eliminate duplicate product carbocations. The output of our simulation is a carbocationic reaction network, where nodes are intermediates and edges are reactions (Fig 3; it should be noted that not all of the intermediates and edges are shown, for simplicity).

To validate our code, we designed an alkane carbocation enumeration experiment for C_5_-C_10_, where linear alkane carbocations are used as the reactants (details see Methods). We expect that the output will contain all alkane carbocation isomers. We then manually drew all the carbocationic isomers for C_5_-C_10_ and compared with the output of our code. As expected, consistent results are obtained (S2 Table).

### Known versus unknown product skeletons

**Fig 4:**
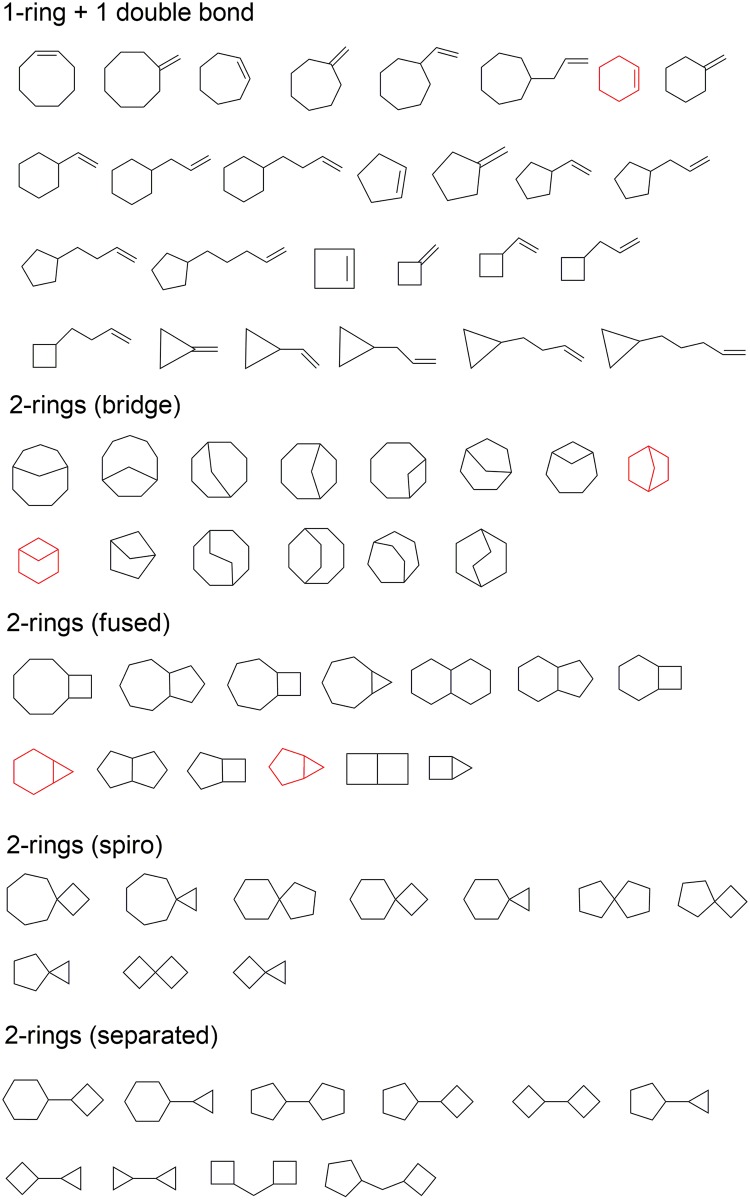
All monoterpene skeletons identified by iGen. Red skeletons have products associated with EC numbers.

The total number of monoterpene carbocations obtained by our simulation is 18758, connected by 123093 virtual reactions (the number of edges). To organize the chemical space of carbocations in a simple manner, we define skeletons for the neutralized carbocation with the saturated alkyl side chains removed (Fig 1). When we group carbocations in this way, 74 cyclized skeletons are found. These cyclized skeletons can be divided into five groups: 1) one ring plus one double bond; 2) two rings containing bridged carbons; 3) two fused rings; 4) two rings linked by a spiro carbon; and 5) two separated rings (Fig 4). To date, only five monoterpene skeletons are associated with EC numbers (by IUBMB; see red skeletons in Fig 4 and S3 Table), all of which can be found among the 74 skeletons found by our automated approach.

Interestingly, none of the known skeletons belong to the groups that have two rings joined at a spiro carbon or two separated rings. More broadly, although we cannot claim to have performed an exhaustive search, we have not identified any known natural products for 69 of the skeletons. Do the 5 skeletons with EC numbers have any features that distinguish them from the 69 unobserved skeletons? Are any of these alternative skeletons plausible, in terms of representing backbone structures that might in the future be identified among monoterpene natural products, among the many that undoubtedly remain unidentified at present; or that might be accessible by enzyme engineering?

The stability of carbocations is an important consideration. For example, secondary carbocations are avoided in most of the terpene synthase reactions. To begin to address this issue, albeit in a somewhat simplistic manner, we applied more stringent energy filters in an attempt to eliminate less stable carbocations. As desired, the fraction of secondary carbocations decreased as we made the energy cutoff more stringent (S1 Fig and S1 Table). Specifically, with the original 0 kcal/mol energy cutoff (energies are relative to the geranyl carbocation, in kcal/mol), 48% are secondary carbocations. With -5 and -10 kcal/mol energy cutoffs, the fraction of secondary carbocations decrease to 33% and 16%, respectively. When applying these two more stringent energy cutoffs, the number of cyclic skeletons identified decreased from 74 to 38 and 35 cyclic skeletons, respectively (S2 Fig). Notably, no skeletons containing two separated rings were found, probably because they are unstable.

**Fig 5:**
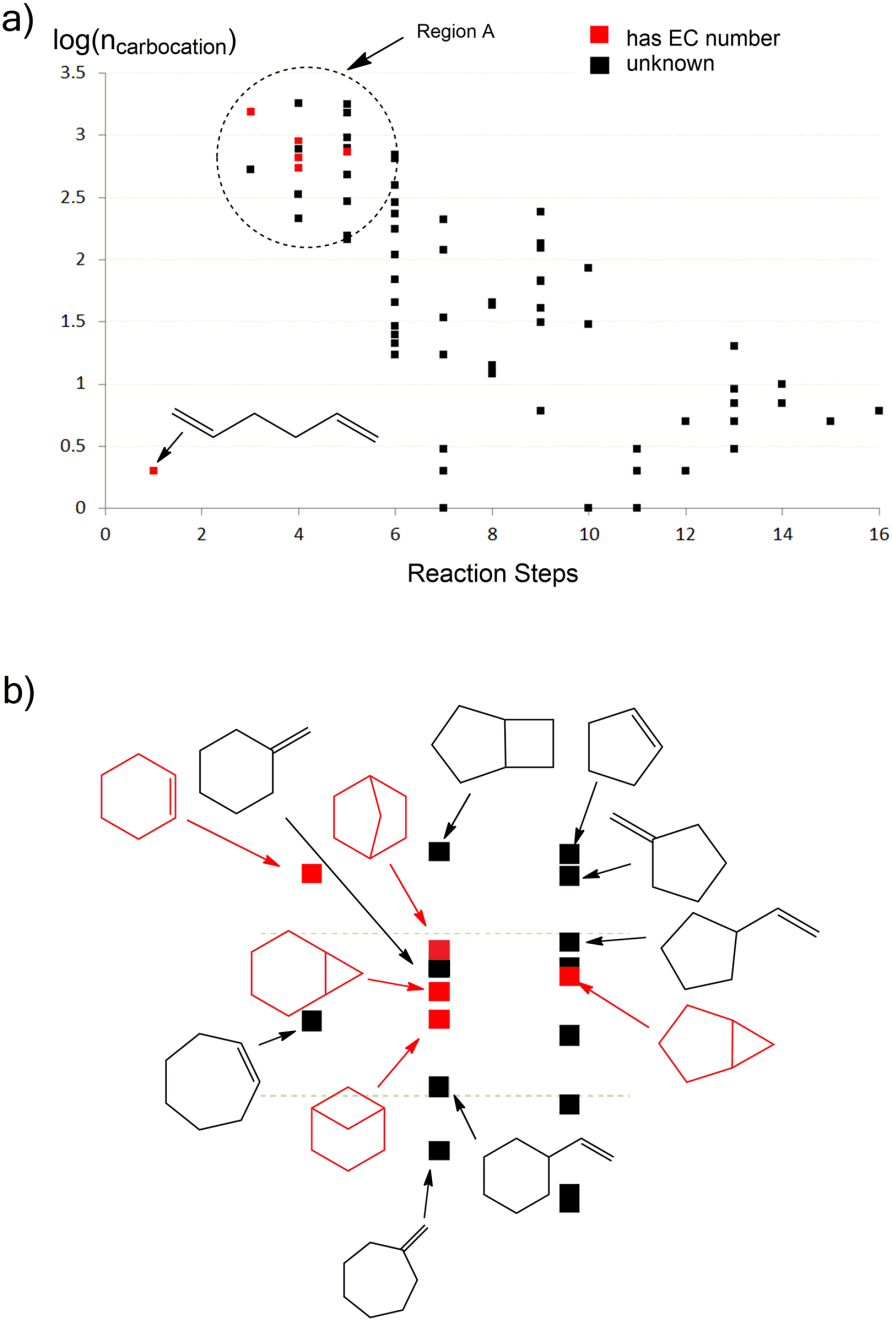
**a) Scatter plot of log(n_carbocation_) versus the number of steps in the shortest reaction route from the linear substrate to the product skeletons**. Red dots indicate skeletons that have products associated with EC numbers. The remaining skeletons (black) have not, to our knowledge, been observed in characterized monoterpenoid natural products. “step 1” is creation of the trans linear carbocation, and “step 2” represents *trans*/*cis* isomerization (which does not create cyclized skeletons). Therefore, cyclic skeletons are generated from “step 3”. **b) Zoom-in view of the circled region in (a), with the structures of the skeletons depicted**. For additional details, see S3 Fig.

Fig 5 maps the skeletons onto two variables, specifically the logarithm of the number of carbocations associated with each skeleton [log(n_carbocation_)], versus the number of reaction steps in the shortest route to obtaining the skeleton from the linear reactant. The number of carbocations associated with a skeleton is largely related to the number of possible substitution patterns and stereoisomers associated with each skeleton. This number is also strongly correlated with the number of reaction steps. The product skeletons associated with known EC numbers (in red) are located primarily in the top-left corner of the plot.

Monoterpene skeletons that can only be accessed through a large number of transformations (5 or greater) do not appear to be represented among known natural products, although more than 5 rearrangement steps are required for the product formation of some sesquiterpenoid synthases, e.g. epi-isozizaene synthase. Seven skeletons are accessible in "step 4" of Fig 5, the step immediately following the first cyclization step. Of these, 3 have associated EC numbers; the remaining 4 skeletons would seem to be excellent candidates for currently uncharacterized monoterpenoid natural products or for enzyme engineering, although we cannot of course prove this. It should be noted that some of the skeletons may not be accessible because high-energy intermediates and transition states are involved, e.g. the methylenecycloheptane skeleton at “step 4” (it is not found in the simulation with -5 kcal/mol energy cutoff).

To explore whether the predicted skeletons are stable compounds, we manually searched the chemical database PubChem [15] (S4 Table). All the skeletons are found, implying that all these predicted skeletons are stable. The top 30 most populated skeletons are shown in S5 Table.

### Visualization of the reaction network

To visualize the complicated carbocation reaction network, we developed a web application called ‘Search C+’ (available at http://carbocation.jacobsonlab.org:8080/; an example query can be found in S4 Fig). Users can search the carbocation virtual library based on chemical similarity [16]. Once a monoterpene carbocation is found, potential reaction routes can be automatically displayed. Users can also identify the neighboring carbocations of a query carbocation in a local network view (the complete network is too large to display).

To predict potential reaction routes for monoterpene carbocations, we performed graph traversal on the obtained carbocation reaction network. Most carbocations can be accessed via multiple reaction routes, and we keep only the shortest route for each precursor carbocation. To predict the best route, one must obtain accurate reaction energies by performing QM/MM or QM cluster calculations in the presence of enzyme [17,18], which is beyond the scope of the current work. Recently, Lobb generated ~1000 C_7_ carbocation intermediates and transition states by searching reaction types similar to this work, followed by geometry optimizations with DFT methods [19]. A similar approach, including explicitly identifying and optimizing transition states, would be valuable for the terpenoid carbocation intermediates considered here, but the computational cost would be rather high at the present time.

Although previous theoretical studies have provided insights into the reaction mechanism for a number of known mono-, sesqui- and diterpenes [6], this is the first computational study to systematically explore the complete chemical space of monoterpenoid carbocations. It should be noted that non-classical carbocations are not considered in our algorithm and only one conformer is retained for each carbocation.

## Discussion

As a critical first step towards enzymatic activity prediction for terpenoid synthases, we have created a computational algorithm to systematically enumerate plausible carbocationic intermediates and the product carbon skeletons that can formed from them. For monoterpenoid synthases (C_10_), we have run many iterations of the algorithm to identify intermediates and product skeletons that can result from enzymatic transformations proceeding through multiple intermediates. The results encompass all monoterpene synthase activities described by EC numbers, as well as other plausible product skeletons that we speculate could be created by one of the many uncharacterized putative terpene synthase enzymes or by engineered enzymes.

It may be possible to systematically explore the chemical space of sesquiterpene cyclases (C_15_) in an analogous manner, although clearly this will be challenging. Recently, a semi-automatic algorithm has been applied to the generation of sesquiterpene carbocations from the humulyl cation (the 1,11-cyclized intermediate) [20]. However, the computational cost of such an algorithm is high, the output of the algorithm seems to consist of less than 200 carbocations, and some of the known carbocations are not explicitly located [20]. Other algorithms [21] without using quantum mechanics may enumerate highly unstable carbocations.

In our on-going work to apply the methods described here to sesquiterpene carbocations, we have already enumerated millions of possible product-precursor structures. Although the methods described here are computationally efficient, the exponential increase in the number of possible carbocations with chain length makes it unlikely that we can perform such a systematic exploration of diterpenoid or larger carbocations. In a previous study [22], the graph-based enumeration of organic small molecules containing C, N, O, S, and halogens was performed for up to 17 heavy atoms, and 166 billion molecules were obtained (without considering stereochemistry).

However, an alternative approach, appropriate for product prediction of terpene cyclases with crystallographic structures (or sufficiently accurate homology models), is to adapt iGen to create carbocations in the active site of an enzyme. The advantage is that one can eliminate "on the fly" those carbocations that do not fit in the site or are electrostatically incompatible, thus reducing the combinatorial explosion. Thus, in principle, the automatic enumeration algorithm may allow the prediction of novel terpenoid skeletons, which was previously impossible [13,14]. As a first proof-of-concept, we have recently used such an approach to facilitate discovery of a novel sesquiterpene synthase [23].

## Methods

### Automatic enumeration of carbocations

The iGen algorithm for systematically enumerating carbocations is illustrated in Fig 2a. The reactant carbocation intermediates (input structures) undergo carbocation rearrangements according to a set of predefined reaction types (Fig 2b; resonance structures are also generated). The input structures can be any carbocations. In the simulations for the monoterpene carbocations, we initiate the calculations with three cyclic carbocation intermediates, i.e., two 1,6-cyclized intermediates, differing in stereochemistry, and a 1,7-cyclized intermediate (Fig 3 shows an example starting from one of the 1,6-cyclized intermediates). The first two reaction steps, i.e. *trans*/*cis* isomerization of the linear carbocation and the cyclization of the cis linear carbocation, are not shown in Fig 3 for simplicity. We use two key iterations to generate all possible products for a given reactant carbocation (S5 Fig): 1) iterations on atoms of the reactant; 2) iterations on reaction types. Atoms of the reactant carbocation are placed in a reactive atom list, except for the carbocation atom and its three bonded atoms. For each atom in the reactive atom list (iterations on atoms), iGen checks whether this atom fits the features for any of the predefined reaction types; for example, if the reactive atom is a carbon atom in a double bond, it fits the reaction type 1 (e.g., iteration 13 in S5 Fig). Virtual reactions are performed by changing the connectivity of the reactant carbocation. The structure-class of the Schrӧdinger software [24], which has built-in functions such as “addBond”, “deleteBond” and “setFormalCharge”, is used to facilitate the molecular connectivity operations.

The resulting carbocations are energy-minimized using molecular mechanics (MM) and quantum mechanics (QM) calculations. The role of the MM minimization is to obtain reasonable geometries of the products after changing the molecular connectivity (S5 Fig). Further semi-empirical QM minimizations, using the RM1 semi-empirical method of the MOPAC package [25], are used to eliminate high-energy carbocations (specific cutoffs described below).

Duplicate carbocations are identified and eliminated by using Simplified Molecular Input Line Entry System (SMILES strings), which describes chemical structures using ASCII strings. The obtained product carbocations then become reactant carbocations in the next round. This process runs repeatedly until no new carbocations can be generated, or other user-defined criteria such as the maximum round number are reached.

The QM energy cutoff is set to 0.0 kcal/mol (relative to the linear reactant GPP cation). For long-range hydride-shift and proton transfer reactions, a C-H distance-cutoff 5.0 Å is used for these two reaction types after Round 5 (long-range hydride shift and proton transfer sometimes occur in enzymatic reactions, mediated by active site residues or water). However, such reactions normally only occur in the first few steps, e.g., 5-epi-aristolochene synthase [26] and selina-4(15),7(11)-diene synthase [27].

iGen is written in Python and takes advantage of the Python API of the Schrӧdinger software [24], which has many built-in functions such as a SMILES string calculator and MM minimizer.

### Conformational sampling and stereochemistry

For carbocations generated in the first five rounds, conformational sampling is performed by using a Monte Carlo sampling approach implemented in the MacroModel software [24]. Each conformer undergoes virtual reactions as described above. We did not perform full conformational sampling for all the carbocations, as this significantly increases computational costs. We expect that generating more conformers may lead to larger numbers of stereo-isomers among the products but not necessarily more product skeletons.

To improve chemical space sampling, we added a ‘stereochemistry module’, which enables the generation of more stereoisomers for a given carbocation conformer. For example, for the conformer described in iteration 1 of S5 Fig, where the H1-C2-C3-C4 dihedral angle is close to zero degrees, it is not clear which stereoisomer is more favorable. In such cases, the ‘stereochemistry module’ generates both stereoisomers via Cartesian coordinate operations. We first calculate the transformation vectors: 1) two orthogonal vectors (with opposite signs) of the plane defined by the sp2 cation atom are calculated; 2) the final position (Cartesian coordinates) of the reactive atom is determined by the orthogonal vector multiplied by a default bond length; 3) the transformation vector is the difference between the coordinates of the final position and the current position of the reactive atom. If the reactive atom is carbon (reaction types 1, 2 and 4), the coordinates of the atoms bonded to this reactive atom will also be changed via the same vectors as the reactive atom. In this work, the dihedral angle range to invoke the ‘stereochemistry module’ is set to be [-45°~+45°].

### Validation test

We performed a validation test by enumerating all possible C_5_-C_10_ alkane carbocation isomers. By running iGen with reaction types 2–4 (alkyl shift, hydride shift and methyl shift) on a linear alkane carbocation, all the isomers of that alkane carbocation will be generated. It should be noted that reaction types 1 and 5 do not apply to alkane carbocations. We then manually drew all possible C_5_-C_10_ alkane carbocations, and compared with the iGen output (S2 Table). QM calculations are not performed in these tests, because many of the alkane carbocations containing -CH_2_^+^ are unstable in the QM calculations.

## Supporting Information

S1 Movie Demonstration of virtual carbocation rearrangement performed by iGen; the sequence of carbocations is hypothetical, and does not correspond to the mechanism of a known terpenoid synthase. (WMV)

S1 Fig Number of a) carbocations and b) secondary carbocations found by iGen, with different energy filters (0 kcal/mol in black, -5 kcal/mol in red and -10 kcal/mol in blue; these energies are all relative to the geranyl carbocation). (TIF)

S2 Fig Monoterpene skeletons found by using different energy filters. Red and blue skeletons were found with -5 kcal/mol; and blue skeletons were found with -10 kcal/mol.
(TIF)

S3 Fig Skeleton details for Fig 5. (TIF)

S4 Fig Visualization of the complex carbocation reaction network with a web application. (TIF)

S5 Fig Illustration of how virtual reactions are performed by iGen. (TIF)

S1 Table Secondary carbocations from simulations with different energy filters. (DOCX)

S2 Table Enumeration of alkane carbocations. (DOCX)

S3 Table Monoterpene skeletons that have EC numbers. (DOCX)

S4 Table Predicted cyclic monoterpene skeletons, their SMILES strings, and URL for the corresponding compounds (using identity search) in PubChem. (DOCX)

S5 Table Top 30 most populated skeletons from computer simulations. (DOCX)
