# Supplementary figures and images for: Defining the Product Chemical Space of Monoterpenoid Synthases
Source: PLoS Comput Biol. 2016 Aug 12;12(8):e1005053. doi: 10.1371/journal.pcbi.1005053 (PMC4982680; doi:10.1371/journal.pcbi.1005053)

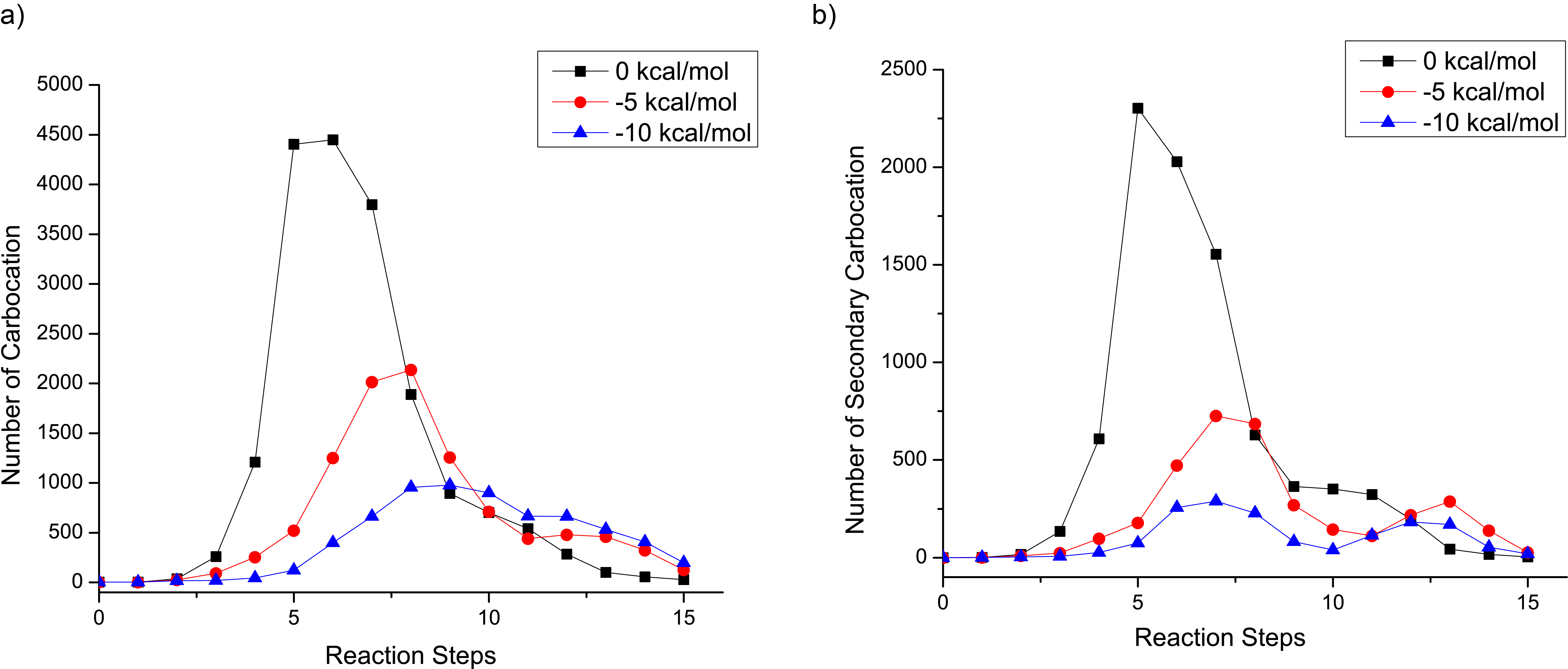

Supplement: S1 Fig — (TIF) [file pcbi.1005053.s002.tif]

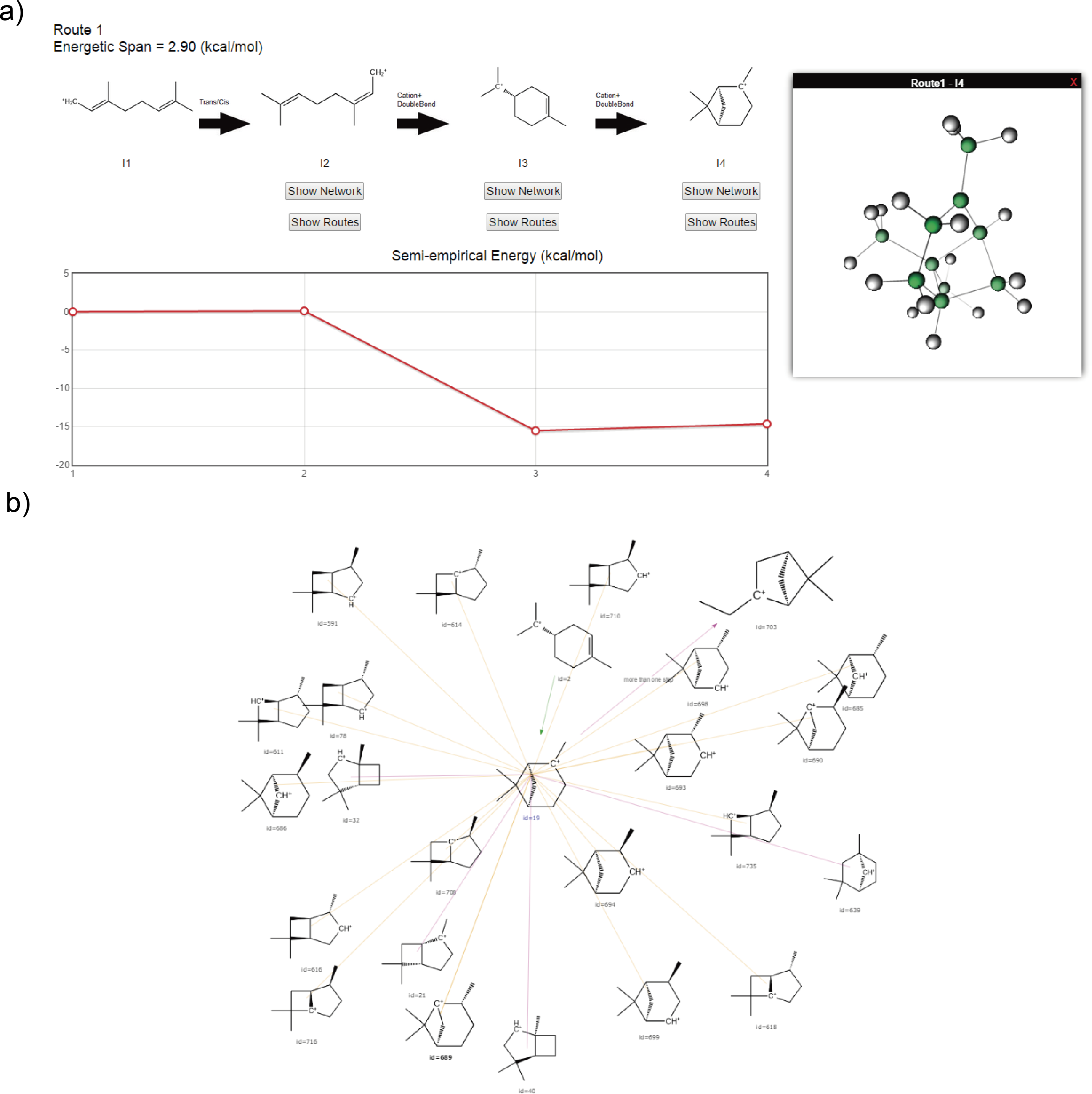

Supplement: S4 Fig — (TIF) [file pcbi.1005053.s005.tif]

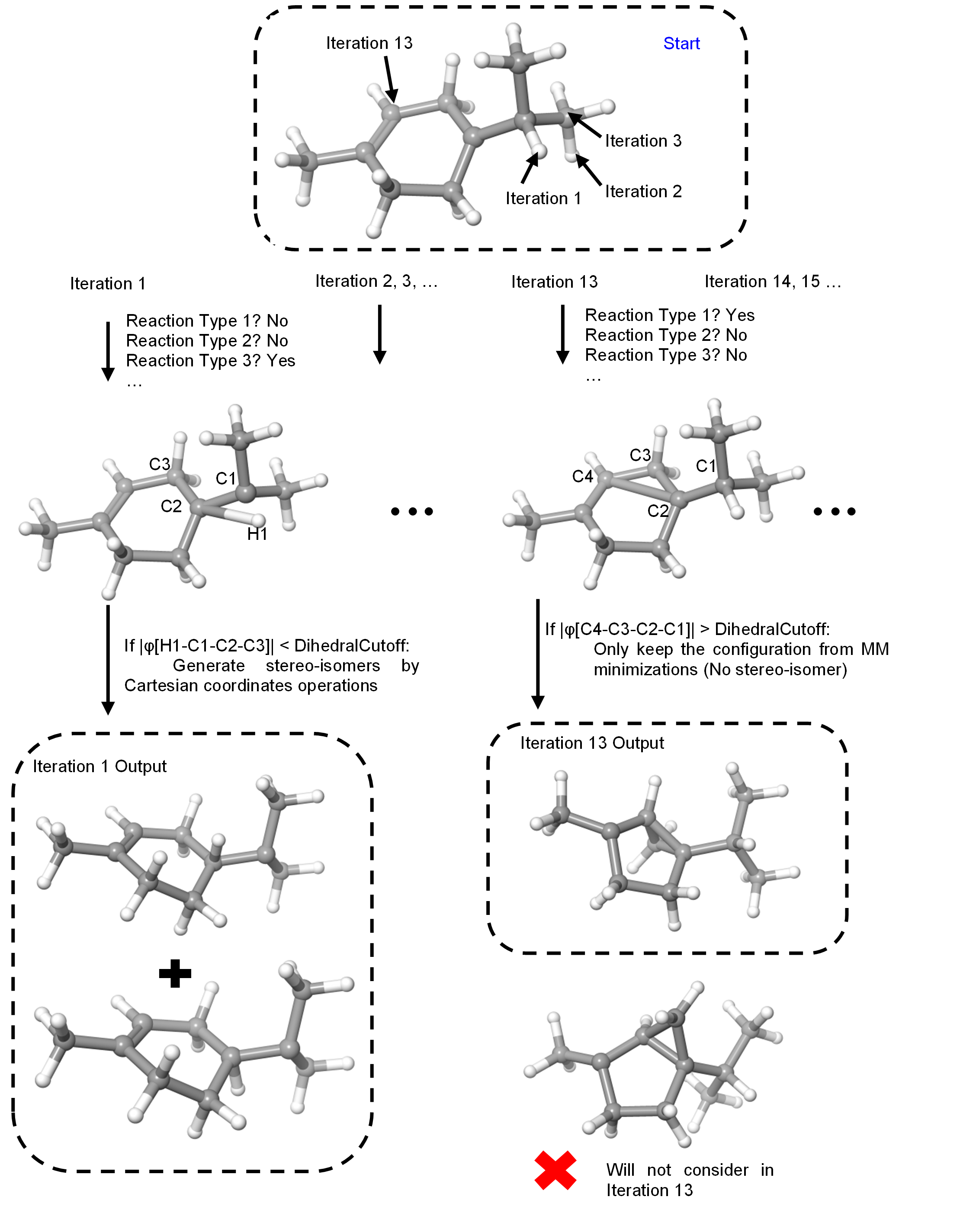

Supplement: S5 Fig — (TIF) [file pcbi.1005053.s006.tif]
