# Supplementary material for: Defining the Product Chemical Space of Monoterpenoid Synthases
Source: PLoS Comput Biol. 2016 Aug 12;12(8):e1005053. doi: 10.1371/journal.pcbi.1005053 (PMC4982680; doi:10.1371/journal.pcbi.1005053)
Supplement: S1 Table — (DOCX) [file pcbi.1005053.s007.docx]

Table S1. Secondary carbocations from simulations with different energy filters

| Energy filter^a^ | Number of carbocations | Number of secondary carbocations^b^ | Fraction of secondary carbocations |
| --- | --- | --- | --- |
| 0 | 18758 | 8649 | 46.1% |
| -5 | 10323 | 3395 | 32.9% |
| -10 | 5599 | 889 | 15.9% |

^a^ energy relative to the geranyl carbocation, in kcal/mol

^b^ without allylic carbocations
