# Supplementary material for: Defining the Product Chemical Space of Monoterpenoid Synthases
Source: PLoS Comput Biol. 2016 Aug 12;12(8):e1005053. doi: 10.1371/journal.pcbi.1005053 (PMC4982680; doi:10.1371/journal.pcbi.1005053)
Supplement: S2 Table — (DOCX) [file pcbi.1005053.s008.docx]

Table S2. Enumeration of alkane carbocations

| Number of Carbon atoms | Number of carbocations generated by iGen^a^ | | | | | Count Manually^b^ |
| --- | --- | --- | --- | --- | --- | --- |
|  | Run 1 | Run 2 | Run 3 | Run 4 | Run 5 |  |
| C5 | 9 | 9 | 9 | 9 | 9 | 9 |
| C6 | 21 | 21 | 21 | 21 | 21 | 21 |
| C7 | 55 | 55 | 55 | 55 | 55 | 55 |
| C8 | 144 | 144 | 144 | 144 | 144 | 144 |
| C9 | 395 | 395 | 395 | 395 | 395 | 395 |
| C10 | 1098 | 1098 | 1098 | 1098 | 1098 | 1098 |

^a^ Five runs from different conformations of the linear carbocation

^b^ c.f. Figure S5
