# Supplementary material for: Defining the Product Chemical Space of Monoterpenoid Synthases
Source: PLoS Comput Biol. 2016 Aug 12;12(8):e1005053. doi: 10.1371/journal.pcbi.1005053 (PMC4982680; doi:10.1371/journal.pcbi.1005053)
Supplement: S3 Table — (DOCX) [file pcbi.1005053.s009.docx]

Table S3. Cyclic monoterpene skeletons that have EC numbers

| Skeletons associated with EC numbers (C_10_ and C_11_) | | EC number |
| --- | --- | --- |
| 1 |  | 4.2.3.20, 4.2.3.108^a^, 4.2.3.112, 4.2.3.113, 4.2.3.16, 4.2.3.111, 4.2.3.52, 4.2.3.114, 4.2.3.115 |
| 2 |  | 4.2.3.121, 4.2.3.122 |
| 3 |  | 4.2.3.10, 5.5.1.8, 4.2.3.116, 5.5.1.22, 4.2.3.105, 4.2.3.117, 4.2.3.118 (C_11_) |
| 4 |  | 4.2.3.109, 4.2.3.110 |
| 5 | ^^ | 4.2.3.107^b^ |

^a^The skeletons of 1,8-cineole (EC 4.2.3.108) are assigned based on product precursor carbocation, rather than the final product (see Fig. 1).

^b^The final product skeleton of (+)-car-3-ene (EC 4.2.3.107) is used, and the product precursor carbocation skeleton is the first skeleton in this table.
