# Supplementary material for: Defining the Product Chemical Space of Monoterpenoid Synthases
Source: PLoS Comput Biol. 2016 Aug 12;12(8):e1005053. doi: 10.1371/journal.pcbi.1005053 (PMC4982680; doi:10.1371/journal.pcbi.1005053)
Supplement: S4 Table — (DOCX) [file pcbi.1005053.s010.docx]

Table S4. Predicted cyclic monoterpene skeletons, their SMILES strings, and URL for the corresponding compounds (using identity search) in PubChem

|  | Skeleton | SMILES String | URL for identical compound in PubChem |
| --- | --- | --- | --- |
| 1 |  | C(CCCC1)C=CC1 | https://pubchem.ncbi.nlm.nih.gov/compound/13609 |
| 2 |  | C=C1CCCCCCC1 | https://pubchem.ncbi.nlm.nih.gov/compound/137991 |
| 3 | 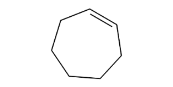 | C1C=CCCCC1 | https://pubchem.ncbi.nlm.nih.gov/compound/12363 |
| 4 |  | C=C1CCCCCC1 | https://pubchem.ncbi.nlm.nih.gov/compound/137620 |
| 5 |  | C=CC1CCCCCC1 | https://pubchem.ncbi.nlm.nih.gov/compound/21530829 |
| 6 |  | C=CCC1CCCCCC1 | https://pubchem.ncbi.nlm.nih.gov/compound/17947502 |
| 7 | 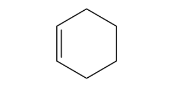 | C1C=CCCC1 | https://pubchem.ncbi.nlm.nih.gov/compound/8079 |
| 8 | 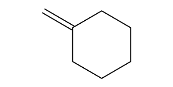 | C1CCCCC1=C | https://pubchem.ncbi.nlm.nih.gov/compound/14502 |
| 9 | 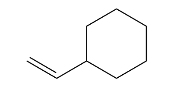 | C1CCCCC1C=C | https://pubchem.ncbi.nlm.nih.gov/compound/12757 |
| 10 | 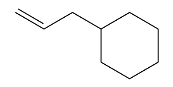 | C1CC(CCC1)CC=C | https://pubchem.ncbi.nlm.nih.gov/compound/75027 |
| 11 |  | C=CCCC1CCCCC1 | https://pubchem.ncbi.nlm.nih.gov/compound/523967 |
| 12 | 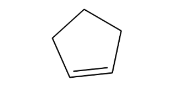 | C1=CCCC1 | https://pubchem.ncbi.nlm.nih.gov/compound/8882 |
| 13 | 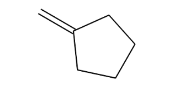 | C=C1CCCC1 | https://pubchem.ncbi.nlm.nih.gov/compound/73714 |
| 14 | 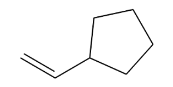 | C1CCCC1C=C | https://pubchem.ncbi.nlm.nih.gov/compound/77345 |
| 15 | 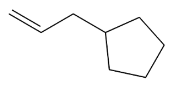 | C=CCC1CCCC1 | https://pubchem.ncbi.nlm.nih.gov/compound/77059 |
| 16 | 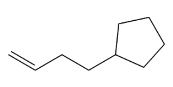 | C1C(CCC1)CCC=C | https://pubchem.ncbi.nlm.nih.gov/compound/12702717 |
| 17 |  | C=CCCCC1CCCC1 | https://pubchem.ncbi.nlm.nih.gov/compound/53931654 |
| 18 | 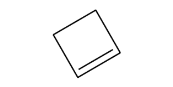 | C1=CCC1 | https://pubchem.ncbi.nlm.nih.gov/compound/69972 |
| 19 | 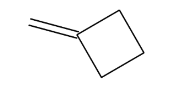 | C=C1CCC1 | https://pubchem.ncbi.nlm.nih.gov/compound/14262 |
| 20 | 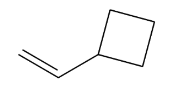 | C=CC1CCC1 | https://pubchem.ncbi.nlm.nih.gov/compound/137655 |
| 21 | 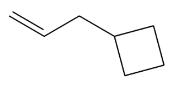 | C=CCC1CCC1 | https://pubchem.ncbi.nlm.nih.gov/compound/22598190 |
| 22 | 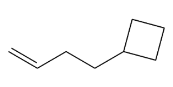 | C1CC(C1)CCC=C | https://pubchem.ncbi.nlm.nih.gov/compound/53908463 |
| 23 | 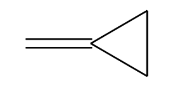 | C=C1CC1 | https://pubchem.ncbi.nlm.nih.gov/compound/80245 |
| 24 | 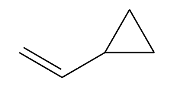 | C=CC1CC1 | https://pubchem.ncbi.nlm.nih.gov/compound/136498 |
| 25 | 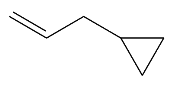 | C1CC1CC=C | https://pubchem.ncbi.nlm.nih.gov/compound/524670 |
| 26 |  | C=CCCC1CC1 | https://pubchem.ncbi.nlm.nih.gov/compound/522662 |
| 27 |  | C=CCCCC1CC1 | https://pubchem.ncbi.nlm.nih.gov/compound/23448194 |
| 28 |  | C1CCC12CCCCCC2 | https://pubchem.ncbi.nlm.nih.gov/compound/12651161 |
| 29 |  | C1CC12CCCCCC2 | https://pubchem.ncbi.nlm.nih.gov/compound/5256394 |
| 30 |  | C1CCCC12CCCCC2 | https://pubchem.ncbi.nlm.nih.gov/compound/135982 |
| 31 | 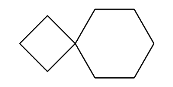 | C1C[C]2(CC[C]1)C[C]C2 | https://pubchem.ncbi.nlm.nih.gov/compound/12651160 |
| 32 | 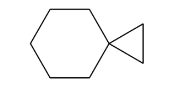 | C1C2(CCCC1)CC2 | https://pubchem.ncbi.nlm.nih.gov/compound/135988 |
| 33 | 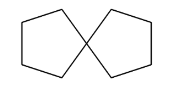 | C1C2(CCC1)CCCC2 | https://pubchem.ncbi.nlm.nih.gov/compound/78959 |
| 34 | 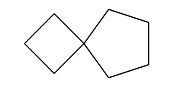 | C1C2(CCC1)CCC2 | https://pubchem.ncbi.nlm.nih.gov/compound/135980 |
| 35 | 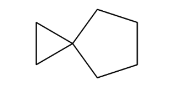 | C1CC12CCCC2 | https://pubchem.ncbi.nlm.nih.gov/compound/12657448 |
| 36 | 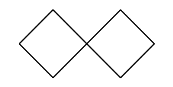 | C1CCC12CCC2 | https://pubchem.ncbi.nlm.nih.gov/compound/20277173 |
| 37 | 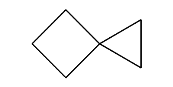 | C1CC2(C1)CC2 | https://pubchem.ncbi.nlm.nih.gov/compound/135974 |
| 38 |  | C1CCC(CC12)CCCC2 | https://pubchem.ncbi.nlm.nih.gov/compound/12592588 |
| 39 |  | C12CC(CC1)CCCCC2 | https://pubchem.ncbi.nlm.nih.gov/compound/13115379 |
| 40 | 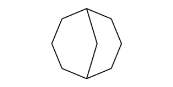 | C1C2CCCC(CCC1)C2 | https://pubchem.ncbi.nlm.nih.gov/compound/12592588 |
| 41 | 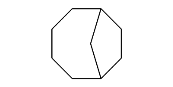 | C1C2CCC(CCC1)C2 | https://pubchem.ncbi.nlm.nih.gov/compound/12556661 |
| 42 | 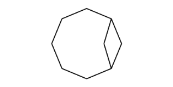 | C1CCC2CC(CCC1)C2 | https://pubchem.ncbi.nlm.nih.gov/compound/23564203 |
| 43 | 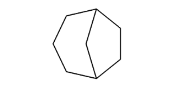 | C12CCCC(C1)CC2 | https://pubchem.ncbi.nlm.nih.gov/compound/260716 |
| 44 | 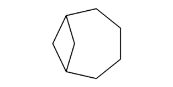 | C1C2CCCCC1C2 | https://pubchem.ncbi.nlm.nih.gov/compound/138914 |
| 45 | 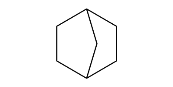 | C12CCC(C1)CC2 | https://pubchem.ncbi.nlm.nih.gov/compound/9233 |
| 46 | 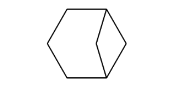 | C12CCCC(C1)C2 | https://pubchem.ncbi.nlm.nih.gov/compound/10129 |
| 47 | 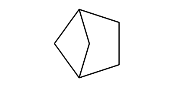 | C12CCC(C1)C2 | https://pubchem.ncbi.nlm.nih.gov/compound/136122 |
| 48 |  | C12CCC(CCC1)CCC2 | https://pubchem.ncbi.nlm.nih.gov/compound/13074880 |
| 49 |  | C12CCC(CC1)CCCC2 | https://pubchem.ncbi.nlm.nih.gov/compound/12659396 |
| 50 | 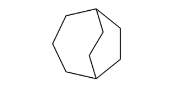 | C12CCC(CCC1)CC2 | https://pubchem.ncbi.nlm.nih.gov/compound/136111 |
| 51 | 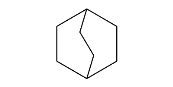 | C12CCC(CC1)CC2 | https://pubchem.ncbi.nlm.nih.gov/compound/9235 |
| 52 |  | C1CC(C12)CCCCCC2 | https://pubchem.ncbi.nlm.nih.gov/compound/524795 |
| 53 |  | C1CCC(C12)CCCCC2 | https://pubchem.ncbi.nlm.nih.gov/compound/138555 |
| 54 | 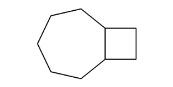 | C1C2C(CCCC1)CC2 | https://pubchem.ncbi.nlm.nih.gov/compound/6427700 |
| 55 | 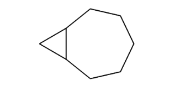 | C12CC1CCCCC2 | https://pubchem.ncbi.nlm.nih.gov/compound/136123 |
| 56 |  | C1CCCC(C12)CCCC2 | https://pubchem.ncbi.nlm.nih.gov/compound/7044 |
| 57 | 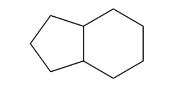 | C1C2C(CCC1)CCC2 | https://pubchem.ncbi.nlm.nih.gov/compound/10325 |
| 58 | 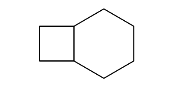 | C1CCCC2C1CC2 | https://pubchem.ncbi.nlm.nih.gov/compound/136091 |
| 59 | 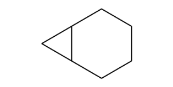 | C1CCCC2C1C2 | https://pubchem.ncbi.nlm.nih.gov/compound/9245 |
| 60 | 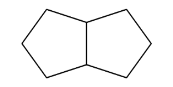 | C1CCC(C12)CCC2 | https://pubchem.ncbi.nlm.nih.gov/compound/136508 |
| 61 | 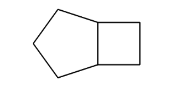 | C1CCC2C1CC2 | https://pubchem.ncbi.nlm.nih.gov/compound/520348 |
| 62 | 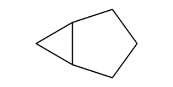 | C1CCC2C1C2 | https://pubchem.ncbi.nlm.nih.gov/compound/67510 |
| 63 | 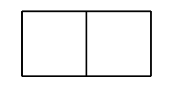 | C1CC(C12)CC2 | https://pubchem.ncbi.nlm.nih.gov/compound/135989 |
| 64 | 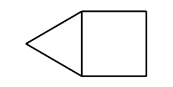 | C1C2C(C1)C2 | https://pubchem.ncbi.nlm.nih.gov/compound/9101 |
| 65 |  | C1CCC1C2CCCCC2 | https://pubchem.ncbi.nlm.nih.gov/compound/23539700 |
| 66 |  | C1CC1C2CCCCC2 | https://pubchem.ncbi.nlm.nih.gov/compound/141725 |
| 67 |  | C1CCCC1C2CCCC2 | https://pubchem.ncbi.nlm.nih.gov/compound/15417 |
| 68 |  | C1CCC1C2CCCC2 | https://pubchem.ncbi.nlm.nih.gov/compound/18512697 |
| 69 |  | C1CCC1C2CCC2 | https://pubchem.ncbi.nlm.nih.gov/compound/548945 |
| 70 |  | C1CC1C2CCCC2 | https://pubchem.ncbi.nlm.nih.gov/compound/17884104 |
| 71 |  | C1CC1C2CCC2 | https://pubchem.ncbi.nlm.nih.gov/compound/15024801 |
| 72 |  | C1CC1C2CC2 | https://pubchem.ncbi.nlm.nih.gov/compound/138564 |
| 73 |  | C1CCC1CC2CCC2 | https://pubchem.ncbi.nlm.nih.gov/compound/20397520 |
| 74 |  | C1CCC1CC2CCCC2 | https://pubchem.ncbi.nlm.nih.gov/compound/57000714 |
