# Supplementary material for: Defining the Product Chemical Space of Monoterpenoid Synthases
Source: PLoS Comput Biol. 2016 Aug 12;12(8):e1005053. doi: 10.1371/journal.pcbi.1005053 (PMC4982680; doi:10.1371/journal.pcbi.1005053)
Supplement: S5 Table — (DOCX) [file pcbi.1005053.s011.docx]

Table S5. Top 30 most populated skeletons from computer simulations^a^

| Rank | Skeleton | Number of carbocations | Reaction steps of the shortest route | Rank | Skeleton | Number of carbocations | Reaction steps of the shortest route |
| --- | --- | --- | --- | --- | --- | --- | --- |
| 1 |  | 1807 | 4 | 16 |  | 397 | 6 |
| 2 |  | 1779 | 5 | 17 |  | 335 | 4 |
| 3 |  | 1548 | 3 | 18 |  | 295 | 5 |
| 4 |  | 1515 | 5 | 19 |  | 286 | 6 |
| 5 |  | 952 | 5 | 20 |  | 241 | 9 |
| 6 |  | 896 | 4 | 21 |  | 234 | 6 |
| 7 |  | 797 | 4 | 22 |  | 232 | 6 |
| 8 |  | 781 | 5 | 23 |  | 212 | 4 |
| 9 |  | 740 | 5 | 24 |  | 210 | 7 |
| 10 |  | 702 | 6 | 25 |  | 175 | 6 |
| 11 |  | 662 | 4 | 26 |  | 155 | 5 |
| 12 |  | 654 | 6 | 27 |  | 144 | 5 |
| 13 |  | 553 | 4 | 28 |  | 135 | 9 |
| 14 |  | 543 | 3 | 29 |  | 123 | 9 |
| 15 |  | 481 | 5 | 30 |  | 120 | 7 |

^a^ red skeletons have products associated with EC numbers,
